# Reply: “Letter to the Editor Re: Billeaud et al. *Nutrients* 2018, *10*, 690”

**DOI:** 10.3390/nu11020406

**Published:** 2019-02-15

**Authors:** Claude Billeaud, Carole Boué-Vaysse, Leslie Couëdelo, Philippe Steenhout, Jonathan Jaeger, Cristina Cruz-Hernandez, Laurent Ameye, Jacques Rigo, Jean-Charles Picaud, Elie Saliba, Nicholas P. Hays, Frédéric Destaillats

**Affiliations:** 1 CIC Pédiatrique 1401 CHU, 33000 Bordeaux, France; claude.billeaud@chu-bordeaux.fr; 2 ITERG, Université de Bordeaux, 33076 Bordeaux, France; c.vaysse@iterg.com (C.B.-V.); l.couedelo@iterg.com (L.C.); 3 Nestlé Health Sciences, 1066 Epalinges, Switzerland; philippe.steenhout@nestle.com; 4 Nestlé Research Centre, 1000 Lausanne, Switzerland; jnthn.jaeger@gmail.com (J.J.); cristina.cruz-hernandez@rdls.nestle.com (C.C.-H.); 5 Nestlé Nutrition R&D, 1800 Vevey, Switzerland; laurent.ameye@nestle.com (L.A.); nicholaspaul.hays@nestle.com (N.P.H.); 6 Department of Neonatology, University of Liège, 4000 Liège, Belgium; j.rigo@ulg.ac.be; 7 Hôpital de la Croix Rousse, Hospices Civils, 69004 Lyon, France; jean-charles.picaud@chu-lyon.fr; 8 Hôpital Clocheville, CHU de Tours, 37004 Tours, France; elie.saliba@univ-tours.fr

**Keywords:** arachidonic acid, docosahexaenoic acid, linoleic acid, fatty acid metabolism, preterm infant nutrition, milk supplements, breast milk

We thank Bernard and colleagues for their careful reading and interest in our article *Effects on Fatty Acid Metabolism of a New Powdered Human Milk Fortifier Containing Medium-Chain Triacylglycerols and Docosahexaenoic Acid in Preterm Infants* [1]. Bernard et al. noted that the fatty acid composition of the plasma total phospholipids in our preterm infant subjects was surprisingly different from the expected composition. In particular, the expected composition should equal 11% oleic acid (OA, 18:1 n-9), 20–22% linoleic acid (LA, 18:2 n-6), 10% arachidonic acid (ARA, 20:4 n-6), and 3% docosahexaenoic acid (DHA, 22:6 n-3). We reexamined our results and realized that an erroneous version of Table 4 had been accidentally incorporated into the paper during manuscript revisions. We sincerely apologize for this unfortunate error and regret any subsequent confusion. A corrected version of Table 4 is below. With a phospholipid fatty acid composition of 13.1% OA, 14.1% LA, 9.4% ARA, and 3.6% DHA, these corrected values are more in-line with those expected. Although the proportion of LA still appears somewhat lower than that expected by Bernard et al. [2], our reported values are generally consistent with those reported by some groups for preterm infants at 30 weeks of gestational age and fed for 21 days [3,4,5,6,7]. In accordance with the observations by Bernard et al. [2], the level of LA in plasma phospholipids gradually increased in all the cited studies.

A second point raised by Bernard et al. [2] is that the LA values in plasma triacylglycerols (reported in Table 5) are surprisingly low, but these results are consistent with those reported elsewhere [3].

Finally, we agree with the remaining two points raised by Bernard and colleagues [2]. First, the ARA/DHA of human milk (HM) fortified with the new fortifier (nHMF) is 0.87, which is close to but still lower than the physiological ratio of > 1. The composition of the nHMF was designed to better support the DHA requirements of preterm infants, since the DHA content in HM is widely variable and typically lower in mothers consuming a Western diet [8] whereas, HM content of ARA is generally higher and less variable [9]. Our data shows enriched DHA content of red blood cell phosphatidylethanolamine in infants fed HM fortified with the nHMF supports the appropriateness of the nHMF composition for preterm infant development. Second, we acknowledge that the best insights into lipid metabolism would result from studies using stable isotope methodologies to investigate fatty acid fluxes and kinetics. This methodology was beyond the scope of our published work.

We fully share the perspective of Bernard and colleagues that improving preterm infants’ lipid nutrition is an urgent task, and we are appreciative of the opportunity to provide a corrected Table 4 and to reply to these important questions.

## Figures and Tables

**Table 4 (Corrected):** Fatty acid profile (g/100 g of fatty acids) of plasma total phospholipids in preterm infants receiving human milk fortified with a control (cHMF) or with a new human milk fortifier (nHMF), before and after 21 days of treatment. Estimations of the treatment effect nHMF/cHMF (difference) and the two-sided p-value are given for each fatty acid analyzed in the different lipid compartments.

	cHMF (*n* = 21)				nHMF (*n* = 26)					
	Baseline		After 21 Days		Baseline		After 21 Days		Difference	*p* Value
	Mean	SD	Mean	SD	Mean	SD	Mean	SD		
14:0	0.21	0.14	0.22	0.14	0.24	0.13	0.26	0.13	0.184	0.500
15:0	0.13	0.10	0.18	0.26	0.14	0.08	0.12	0.03	−0.071	0.722
16:0	25.00	5.55	22.75	6.21	26.00	3.58	26.46	2.76	0.180	0.020
16:0 DMA	0.63	0.18	0.70	0.17	0.64	0.16	0.74	0.27	−0.134	0.394
16:1 *n*-7	1.24	0.71	1.05	1.19	1.28	0.69	0.93	0.70	0.209	0.188
16:1 *n*-9	0.32	0.14	0.24	0.15	0.30	0.12	0.25	0.13	0.219	0.117
18:0	14.99	2.90	17.25	4.15	14.51	1.65	15.84	1.63	−0.060	0.203
18:0 DMA	0.33	0.12	0.47	0.37	0.35	0.10	0.36	0.14	−0.402	0.040
18:1 DMA	0.29	0.14	0.32	0.11	0.30	0.09	0.30	0.13	−0.288	0.054
18:1 *n*-7	3.07	0.59	2.36	0.62	3.05	0;89	2.59	0.77	0.122	0.015
18:1 *n*-9	14.04	2.16	11.98	3.37	13.90	3.03	12.45	2.97	0.051	0.286
*trans*-18:1	0.30	0.15	0.42	0.16	0.39	0.13	0.40	0.17	−0.112	0.515
18:2 *n*-6 (LA)	12.90	3.20	14.60	2.53	14.03	2.75	14.69	2.55	0.043	0.452
18:3 *n*-3 (ALA)	0.18	0.14	0.11	0.05	0.18	0.10	0.17	0.08	0.219	0.125
18:3 *n*-6 (GLA)	0.22	0.07	0.17	0.06	0.22	0.06	0.16	0.04	0.030	0.689
20:0	0.36	0.29	0.40	0.31	0.38	0.21	0.41	0.18	0.011	0.945
20:1 *n*-9	0.25	0.08	0.31	0.09	0.27	0.10	0.36	0.12	0.156	0.072
20:2 *n*-6	0.47	0.13	0.67	0.46	0.51	0.11	0.53	0.10	−0.112	0.340
20:3 *n*-6 (DGLA)	3.79	0.89	4.04	1.05	3.46	0.72	3.45	0.68	−0.161	0.041
20:3 *n*-9	2.56	1.46	1.90	1.53	2.12	1.19	2.11	1.51	0.089	0.612
20:4 *n*-6 (ARA)	9.81	2.33	10.63	3.54	9.09	2.03	8.26	2.58	−0.211	0.015
20:5 *n*-3 (EPA)	0.84	0.48	0.70	0.25	0.88	0.32	0.97	0.28	0.318	0.006
22:0	0.46	0.39	0.53	0.53	0.53	0.33	0.57	0.27	0.110	0.567
22:1 *n*-9	0.05	0.03	0.05	0.03	0.05	0.02	0.05	0.02	0.048	0.676
22:4 *n*-6	0.46	0.17	0.49	0.29	0.40	0.13	0.35	0.10	−0.257	0.032
22:5 *n*-3 (*n*-3 DPA)	0.46	0.24	0.53	0.31	0.44	0.12	0.40	0.12	−0.217	0.037
22:5 *n*-6 (*n*-6 DPA)	0.49	0.19	0.53	0.25	0.45	0.25	0.50	0.22	−0.005	0.970
22:6 *n*-3 (DHA)	3.60	1.42	3.96	1.94	3.22	0.93	3.54	1.04	−0.050	0.575
24:0	0.39	0.29	0.53	0.80	0.48	0.28	0.43	0.18	−0.056	0.742
24:1 *n*-9	1.50	1.23	1.47	0.83	1.55	0.98	1.77	0.73	0.191	0.368

Data are presented as geometric mean and geometric standard deviation (SD). ALA, α-linolenic acid; ARA, arachidonic acid; DGLA, dihomo-γ-linolenic acid; DHA, docosahexaenoic acid; DMA, dimethyl acetal; DPA, docosapentaenoic acid; EPA, eicosapentaenoic acid; GLA, γ-linolenic acid; LA, linoleic acid.
